# The Impact of Dependency Burden on Urban Household Health Expenditure and Its Regional Heterogeneity in China: Based on Quantile Regression Method

**DOI:** 10.3389/fpubh.2022.876088

**Published:** 2022-05-04

**Authors:** Xiaocang Xu, Qingqing Wang, Chang Li

**Affiliations:** ^1^School of Economics and Management, Huzhou University, Huzhou, China; ^2^Research Center for Economy of Upper Reaches of the Yangtse River/School of Economics, Chongqing Technology and Business University, Chongqing, China; ^3^Business School, Zhengzhou University, Zhengzhou, China; ^4^School of Economics and Management, Hebei University of Science and Technology, Shijiazhuang, China

**Keywords:** dependency burden, health expenditure, regional heterogeneity, quantile regression method, aging

## Abstract

**Background:**

The aging population has led to a growing health expenditure burden. According to the National Bureau of Statistics of China, the old-age dependency ratio rose from 10.7% in 2003 to 17.8% in 2019, and health expenditure increased from 658.410 billion yuan in 2003 to 5812.191 billion yuan in 2019 in China.

**Methods:**

This paper utilizes the quantile regression method to discuss the influencing factors of health expenditure in urban China based on the China Household Finance Survey (CHFS), especially dependency burden. Moreover, its regional heterogeneity is also compared.

**Results:**

The old-age dependency ratio, age, family size, self-rated health status, and income significantly impact the health expenditure of urban families in the quantile regression of the national sample. Dependency burden and other variables on urban household health expenditure have great regional heterogeneity. The relationship between urban health expenditure and residential areas in western China is more stable than that in eastern and central China.

**Discussion:**

Government should improve the healthcare system suitable for the older adult population as soon as possible. The government of western China should pay more attention to the introduction of professional medical talents and the configuration of precision medical equipment to improve the health system in western China.

## Introduction

The aging population has led to a growing medical and health expenditure burden worldwide. For example, people over the age of 65 increased from 96.92 million in 2003 to 176.03 million in 2019, an increase of 81.62%. The old-age dependency ratio rose from 10.7% in 2003 to 17.8% in 2019, with a growth rate of 66.36% in China (National Bureau of Statistics of China, 2020). As a result, the health expenditure increases too rapidly. Global scholars have given great importance to health expenditure since the 1950s and found that the influencing factors of health expenditure mainly include income level, education level, medical insurance, and so on.

### Income Level and Health Expenditure

The influence of income on health expenditure is mostly positive. For example, Newhouse (1) found that the continuous growth of health expenditure was positively correlated with income in the United States. Ross et al. (2) used 3-year cross-sectional data of the Health and Retirement Study (HRS) to find that the preventive health expenditure of poor workers was lower than that of higher-income workers. Costa-Font et al. (3) concluded that the income elasticity of health expenditure is <1, and Reeves and Kee (4) found that the income elasticity of health expenditure is only 0.5. In China, Hou and Ren (5) used panel data to show that total health expenditure would increase with gross domestic product (GDP) growth. Xu and Chen (6) analyzed the health expenditure of urban and rural residents in 29 provinces of China and concluded that the health expenditure of rural residents was more sensitive to income. Tian and Qu (7), Xu et al. (8), and Xie et al. (9) suggest that income difference is an important reason for the difference in health expenditure between urban and rural residents.

### Education and Health Expenditure

In addition to income level, education level also affects the health needs of residents, which in turn affects health expenditure. Mwabu et al. (10) found that the improvement in education level would enhance health awareness and make them more willing to pay more for health expenditure in Kenya. Hjortsberg (11) found that education level was one of the most important factors that affect the health expenditure of Zambian residents. Sato (12) used the two-digit model to find that the demand for healthcare services is directly proportional to education in Ghana. Mackenbach et al. (13) found that absolute inequalities in acceptable mortality due to healthcare conditions remained stable over time between low and high levels of education, while relative inequalities widened between 1980 and 2010 in 17 European countries. In China, Tian et al. (14) showed that health expenditure is significantly positively correlated with education level, especially in western China. Dai and Li (15) used a spatial autoregressive model to find that the health expenditure of urban residents is negatively correlated with education level. Zhang et al. (16) adopted the dynamic generalized moment estimation method and showed a negative relationship between health expenditure and per capita education years in rural regions.

### Health Insurance and Health Expenditure

Countries around the world hope to improve health insurance to reduce the medical burden on residents. Hurd and McGarry (17) found that older adults with medical insurance have a higher probability of using medical care services. Biro (18) and Kavosi et al. (19) found that medical insurance effectively affects health expenditure in Europe. Azam (20), Wu et al. (21), and Sepehri and Vu (22) all confirmed that urban residents' participation in medical insurance can effectively avoid the persecution of family catastrophic medical expenses. In China, Fang et al. (23) concluded that the health expenditure of families without social medical insurance was significantly higher than that of insured families (23). According to the panel data of the Chinese Longitudinal Healthy Longevity Survey (CLHLS), Wang and Zheng (24) found that the promotion effect of medical insurance on health expenditure had significant differences between urban and rural regions. Xiong and Huang (25) used the Blinder–Oaxaca decomposition model and found that medical insurance policy is an important factor that leads to the differences in health expenditure between urban and rural residents.

### Dependency Burden on the Older Adults and Health Expenditure

Healthcare consumption increases with age due to the dependency burden. Fuchs (26) pointed out that the growth rate of health expenditure for the older adults was higher than GDP. Carreras et al. (27) used a two-part model to prove that aging will cause an increase in health expenditure to a certain extent. In China, Li et al. (28) showed that the health expenditure of residents aged 65 and above increased most drastically in Beijing. Tan et al. (29) showed that families with older adults had significantly higher demand for health services and health expenditure than those without older adults. At the same time, some scholars believe that there is no great relationship between population aging and health expenditure. For example, Barros (30) used the statistical data of organisation for economic co-operation and development (OECD) countries to study the determinants of the increase in total health expenditure and found that aging was not the cause of the increase in total health expenditure.

To sum up, global scholars have discussed the possible influencing factors of health expenditure from different dimensions. Most studies believe that income level, education level, and health insurance impact health expenditure. However, few studies discussed the effect of population aging (represented by dependency burden) on health expenditure or have not reached uniform conclusions. Therefore, this paper tries to use the quantile regression method to discuss the influencing factors of health expenditure in urban China based on the China Household Finance Survey (CHFS), especially dependency burden. Moreover, its regional heterogeneity is also compared. The quantile regression method is used because it can capture the characteristics of urban household health expenditure in different quantiles nationwide and among regions and more accurately describe the influencing factors of health expenditure.

It should be noted that the latest data on CHFS was released in 2017 when we wrote the paper, and its data samples covered 400,011 households and 127,012 individuals in 29 provinces (municipalities and autonomous regions). It involves household demographic characteristics, assets and liabilities, insurance and security, income and expenditure, and other micro-household financial data. Furthermore, the rejection rate of CHFS is low, and the demographic characteristics are very similar to the data published by the National Bureau of Statistics of China.

## Materials and Methods

### Variable Selection

#### Explained Variable—Household Health Expenditure

Household health expenditure is divided by the total population of each household based on the personal ID calculation generated by the tracking of the respondents by sorting out the answers to the CHFS questionnaire “how much did your family spend on healthcare last year.” In the process of data collation, considering the sufficient sample size, the missing value and uncertain invalid data in the original data of family health expenditure are first removed and then divided by the total population of each family to obtain the per capita health expenditure of the family. At the same time, by observing the QQ chart and boxplot of the per capita health expenditure of the family, we can see that the median of the dependent variable is small and has a large outlier. The QQ chart is a curve with asymmetry, which differs greatly from the normal QQ line. In general, the per capita health expenditure of the family in the sample data does not meet the normal distribution. Second, to reduce the volatility of the variable itself, the family per capita health expenditure is logarithmically transformed. The reason for doing this is that the logarithm will not change the nature and correlation of the data, but the scale of variables is compressed, the data are more stable, and the collinearity and heteroscedasticity of the model are weakened.

#### Core Explanatory Variable—Dependency Burden

The key indicator of dependency burden is the old-age dependency ratio. Most studies use the old-age dependency ratio to measure the degree of population aging. Different from the previous literature, this paper calculates the proportion of the older adult population (aged 65 and above) to the working-age population (aged 15–64) according to the characteristics of the micro-household database, so as to measure the dependency burden for the older adults. Therefore, the higher the old-age dependency ratio is, the more significant part of the value created by the working population will be used for the consumption of the older adults, which will put a heavier dependency burden on the society and reduce the income of the working population.

#### Other Explanatory Variables

Other explanatory variables include age, education level, marital status, and others. The specific processing process is shown in Table 1.

**Table 1:** Description of each variable.

**Variable types**	**Variable name**	**Abbreviation**	**Variable declaration**
Explained variable	Household health expenditure per capita	lnPE	Take the logarithm of the total health expenditure per urban household in the past year
Core explanatory variable	Dependency burden (old-age dependency ratio)	OR	Proportion of the population aged 65 or above in the working population per household
Other explanatory variables	Age	Age	The age of the head of each household in 2017
	Gender	Gender	Gender of head of household (male = 1; Female = 2)
	Marital status	M	Marital status of head of household (unmarried = 1; Married = 2)
	Education Level	jy	Education level of head of household (no schooling = 0; Primary school = 6; Junior high school = 9; High school = 12; Technical secondary school/Vocational high school =13; Junior college/Higher vocational =15; Bachelor degree =16; Master =19; PhD = 22). The different numbers correspond to the number of years of study at each stage in China.
	Family size	Number	The total number of people per household
	Self-rated health	P	Household head's self-rated physical condition (good =1; General = 2; Poor = 3)
	Household incomes per capita	lnPI	The total income per household in the last year divided by the total number of people in the household. Take the logarithm
	Social medical insurance	YB	No social health insurance = 1; Basic medical insurance for urban workers = 2; Basic medical insurance for urban residents = 3; New rural cooperative medical insurance = 4
	Location	Location	urban= 1, country = 2

### Data Source

The empirical data of this paper are from the household database published by China Household Finance Survey (CHFS), which is a nationwide sample survey designed to collect relevant information about household finance at the micro-level. CHFS is being conducted every 2 years since its inception in 2009. It has successfully carried out random household sampling surveys nationwide for four times in 2011, 2013, 2015, and 2017. This paper selects the data samples that cover 40,011 households and 127,012 individuals in 29 provinces in China Household Finance Survey (2017), involving micro-household financial data such as household demographic characteristics, assets and liabilities, insurance and security, income and expenditure, and so on. Finally, 19,787 households were selected as empirical samples by eliminating the missing value and outliers of the sample data. The rejection rate of CHFS was low and the sample size was abundant, while the missing values and outliers were few. Therefore, deleting them in the case of a large sample size had less effect on the results.

### Empirical Methods and Model Setting

Classical regression such as ordinary least squares (OLS) is focused on “mean regression” and is susceptible to extreme values because the objective function for minimization is residual squared and $\overset{n}{\underset{i=0}{\sum }}{e_{i}}^{2}$. Therefore, Koenker and Bassett (31) put forward the quantile regression method, which uses the residual error absolute value of the weighted average (such as $\overset{n}{\underset{i=0}{\sum }}|e_{i}|$) to minimize the objective function and thus not affected by extreme value and relatively stable. More importantly, quantile regression can provide comprehensive information on different quantiles.

Due to the imbalance between economic development and the allocation of medical resources in China, the health expenditure of most families remains at a low level compared with that of developed countries in Europe and the United States. However, it is not excluded that some families have high health expenditures due to special reasons, which may lead to the uneven distribution of health expenditure allocation. This case will cause errors if mean regression is still used to estimate the overall model. Therefore, the quantile regression method is used, because it can capture the characteristics of urban household health expenditure in different quantiles nationwide and among regions and more accurately describe the influencing factors of health expenditure. The model is set as Equation 1:


(1) $$\begin{array}{l} \begin{array}{c} lnPE_{i}=\beta_{0}+\beta_{1}OR_{i}+\beta_{2}age_{i}+\beta_{3}gender_{i}+\beta_{4}M_{i}+\beta_{5}jy_{i} \end{array}\\ \begin{array}{c} +\beta_{6}number_{i}+\beta_{7}P_{i}+\beta_{8}PI_{i}+\;\beta_{9}YB_{i}+\;\beta_{10}location_{i}+\varepsilon_{i} \end{array} \end{array}$$

where *lnPE*_*i*_ is the logarithm of the per capita health expenditure of the urban household i, *OR*_*i*_is the old-age dependency ratio of the urban household i; *age*_*i*_ is the age of the head of household i; *gender*_*i*_ indicates the gender of the head of household i. *M*_*i*_ is the marital status of the head of household i; *jy*_*i*_ represents the educational level of the head of household i; *number*_*i*_ is the size of the household i. *P*_*i*_ represents the overall self-rated health status of the household i (including head of household); *PI*_*i*_ represents the per capita income of household i, *YB*_*i*_ represents the type of social medical insurance, and ε_*i*_ is the random interference term.

## Results

### Descriptive Statistics

To better display the distribution characteristics of variables in the model, a descriptive summary of relevant variables in nationwide, eastern China, western China, and central China is shown in Table 2. Eastern China includes Beijing, Tianjin, Hebei, Liaoning, Shanghai, Jiangsu, Zhejiang, Fujian, Shandong, Guangdong, and Hainan; central China includes Shanxi, Jilin, Heilongjiang, Anhui, Jiangxi, Henan, Hubei, and Hunan; western China includes Sichuan, Chongqing, Guizhou, Yunnan, Tibet, Shaanxi, Gansu, Qinghai, Ningxia, Xinjiang, Guangxi, and Inner Mongolia. The relevant data of Hong Kong, Macao, Taiwan, Tibet, and Xinjiang Uygur Autonomous Region are not included in the database, so they are not included in the analysis.

**Table 2:** Summary statistics of variables.

**Region of sample**	**Variables**	**Obs**	**Mean**	**Std. Dev**.	**Min**	**Max**
Nationwide	lnPE	19,787	6.904	1.726	−1.609	13.305
	OR	19,787	0.251	0.373		
	age	19,787	55.688	13.939	18	117
	jy	19,787	9.233	4.167		22
	number	19,787	3.421	1.825		23
	lnPI	19,787	9.504	1.467	−3.352	14.732
Eastern China	lnPE	11,018	6.915	1.718	−1.609	13.305
	OR	11,018	0.247	0.37		
	age	11,018	55.537	13.84	18	117
	jy	11,018	9.301	4.128		22
	number	11,018	3.414	1.787		22
	lnPI	11,018	9.501	1.469	−2.842	14.732
Central China	lnPE	4,439	6.878	1.742		12.899
	OR	4,439	0.262	0.381		
	age	4,439	56.15	14.186	18	100
	jy	4,439	9.077	4.233		22
	number	4,439	3.413	1.853		23
	lnPI	4,439	9.484	1.481	−3.352	14.262
Western China	lnPE	4,330	6.903	1.73	0.511	12.569
	OR	4,330	0.249	0.371		
	age	4,330	55.599	13.927	20	93
	jy	4,330	9.221	4.196		22
	number	4,330	3.445	1.891		23
	lnPI	4,330	9.531	1.448	−2.436	14.527

In Table 2, we can see the general characteristics of each continuous variable:

Among the three regions, the average logarithm of the per capita health expenditure of urban families in eastern China is RMB 6.915 yuan, exceeding the national average (RMB 6.904 yuan), whereas the mean of the central and western China is RMB 6.878 and 6.903 yuan, respectively, lower than the average of the national sample family per capita health expenditure. Therefore, the average value of explained variables is the highest in eastern China, indicating that eastern families' overall level of per capita health expenditure is higher.

The mean value of the old-age dependency ratio showed the trend of central China greater National greater western China greater eastern China, and the mean value from high to low was 0.262, 0.251, 0.249, and 0.247. At the same time, by comparing the standard deviation of the old-age dependency ratio between the national sample and each region, it can be seen that the aging degree of the samples in each region is similar to a small gap, indicating that the old-age dependency ratio gap between families is reasonable and the sample selection is representative. In addition, the mean of family size, education level, and age in the three regions are very close to the national average. According to the per capita income of urban families after logarithm, the eastern region has the best economic development among the three regions. Therefore, the maximum sample value of per capita health expenditure in the eastern region is 14.732, which is higher than that in the central region (14.262) and western region (14.527). However, the mean of per capita household income showed a trend of western region > eastern region > central region, with the maximum mean of 9.531 in the western region and the minimum mean of 9.484 in the central region, and only the mean of the western region exceeded the national average (9.504).

### Empirical Results of Quantile Regression Model

In this paper, the explained variables were divided into families with different levels of health expenditure, with quantile boundaries of 10, 25, 50, 75, and 90%, respectively.

#### Nationwide

As can be seen from Table 3, the old-age dependency ratio, age, family size, self-rated health status, and per capita family income all significantly impact the explained variables at each subpoint of per capita family health expenditure. Gender, marriage, education level, and place of residence significantly influence the sample range of per capita health expenditure of the family, whereas the influence of social medical insurance is complicated due to various categories.

**Table 3:** National sample quantile regression results.

**Variables**	**QR_10**	**QR_25**	**QR_50**	**QR_75**	**QR_90**
OR	0.804^***^	0.856^***^	0.624^***^	0.588^***^	0.626^***^
	(0.096)	(0.078)	(0.067)	(0.076)	(0.097)
age	0.006^***^	0.011^***^	0.014^***^	0.014^***^	0.012^***^
	(0.002)	(0.002)	(0.002)	(0.002)	(0.003)
gender_2	0.172^***^	0.255^***^	0.139^***^	0.152^***^	0.105
	(0.045)	(0.056)	(0.048)	(0.053)	(0.068)
M_2	0.188^***^	0.262^***^	0.117^**^	0.095	0.047
	(0.043)	(0.061)	(0.057)	(0.074)	(0.105)
jy	0.023^***^	0.021^***^	0.008	−0.001	0.005
	(0.005)	(0.001)	(0.006)	(0.006)	(0.008)
number	−0.110^***^	−0.113^***^	−0.121^***^	−0.112^***^	−0.116^***^
	(0.008)	(0.013)	(0.010)	(0.013)	(0.016)
P_2	0.370^***^	0.424^***^	0.428^***^	0.325^***^	0.213^***^
	(0.041)	(0.048)	(0.041)	(0.047)	(0.056)
P_3	0.948^***^	1.141^***^	1.078^***^	0.886^***^	0.786^***^
	(0.068)	(0.059)	(0.050)	(0.056)	(0.088)
lnPI	0.113^***^	0.120^***^	0.114^***^	0.090^***^	0.074^***^
	(0.015)	(0.015)	(0.016)	(0.019)	(0.025)
YB_2	0.418^***^	0.479^***^	0.499^***^	0.358^***^	0.223^*^
	(0.113)	(0.092)	(0.106)	(0.110)	(0.134)
YB_3	0.082	0.187^*^	0.274^**^	0.150	0.158
	(0.142)	(0.105)	(0.111)	(0.119)	(0.142)
YB_4	−0.098	−0.041	0.142	0.036	−0.133
	(0.106)	(0.087)	(0.104)	(0.106)	(0.131)
location_2	−0.075	−0.148^**^	−0.271^***^	−0.306^***^	−0.251^***^
	(0.050)	(0.058)	(0.053)	(0.060)	(0.075)
Constant	2.906^***^	3.289^***^	4.577^***^	6.149^***^	7.506^***^
	(0.213)	(0.208)	(0.220)	(0.259)	(0.329)
Observations	19,787	19,787	19,787	19,787	19,787

Robust standard errors in parentheses ^***^p < 0.01, ^**^p < 0.05, ^*^p < 0.1.

*The numbers in brackets below the regression coefficients represent robust standard error*.

The old-age dependency ratio has a significant effect on the per capita health expenditure of the family. With the increase of quantile (0.1 → 0.25 → 0.5 → 0.75 → 0.9), the quantile regression coefficient of the old-age dependency ratio showed a U-shaped trend of rising first and then decreasing (0.096 → 0.078 → 0.067 → 0.076 → 0.097). The results show that the old-age dependency rate has the greatest impact on the health expenditure of families with lower health expenditure. At the same time, the standard error of the estimated coefficient decreased first and then increased (0.079 → 0.065 → 0.045 → 0.05 → 0.067), indicating that the estimation of the quantile regression coefficient at both ends of the conditional distribution is not accurate, but the difference in accuracy is small. Therefore, population aging has a greater impact on families with lower levels of health expenditure in China and a smaller impact on families with higher levels of health expenditure.

The influence of age on family health expenditure was significantly positive at each subsite of the national sample. The standard error of the estimated coefficients is consistent, indicating that the impact of age on family health expenditure is relatively stable. With the increase of age, health expenditure increases steadily. Female-headed households have a higher level of health expenditure per capita than the male-headed sample. Married people have a significant positive impact on health expenditure at 0.1, 0.25, and 0.5 subpoints, and the family with lower health expenditure has the greatest impact. The education level of residents is only a significant variable at 0.1 and 0.25 subpoints and has a similar effect on families with low health expenditure, with no significant difference. The regression results of family size show that health expenditure is inversely correlated with the total population, and the health expenditure of a family decreases by 11–12% with each additional family member.

The influence of self-rated health status on the per capita health expenditure of the family is more prominent among all the influencing factors, especially in the sample group with poor self-rated health status. Compared with the residents with good self-rated health status, the impact of the sample with average self-rated health status on the per capita health expenditure of the family shows an inverted “U” trend, which increases first and then decreases. Therefore, when residents self-rated their health status as average, households with moderate health expenditure changed the most, accounting for 42.8%. Meanwhile, poor self-rated health status was the most prominent influencing factor. The standard error of the estimated coefficient showed a U-shaped trend of decreasing first and then increasing (0.068 → 0.059 → 0.050 → 0.056 → 0.088), indicating that the accuracy of the distribution estimation of the household per capita health expenditure is slightly lower.

The effect of social medical insurance and residence on per capita health expenditure is not uniform. First of all, taking the sample group without any social medical insurance as a reference, urban employment insurance can significantly increase health expenditure and have a greater promotion effect on health expenditure with medium and low levels of health expenditure. Place of residence has no significant effect on the per capita health expenditure of families with a low level of health expenditure. The impact of residence on the per capita health expenditure of families at other subloci generally showed an inverted U-shaped trend of rising first and then decreasing. From 0.25 subloci to 0.9 subloci, the regression coefficients were −0.148,−0.271,−0.306, and−0.251, respectively, that is, compared with families living in urban areas. The per capita health expenditure of households in rural areas has decreased significantly.

**Figure 1:**
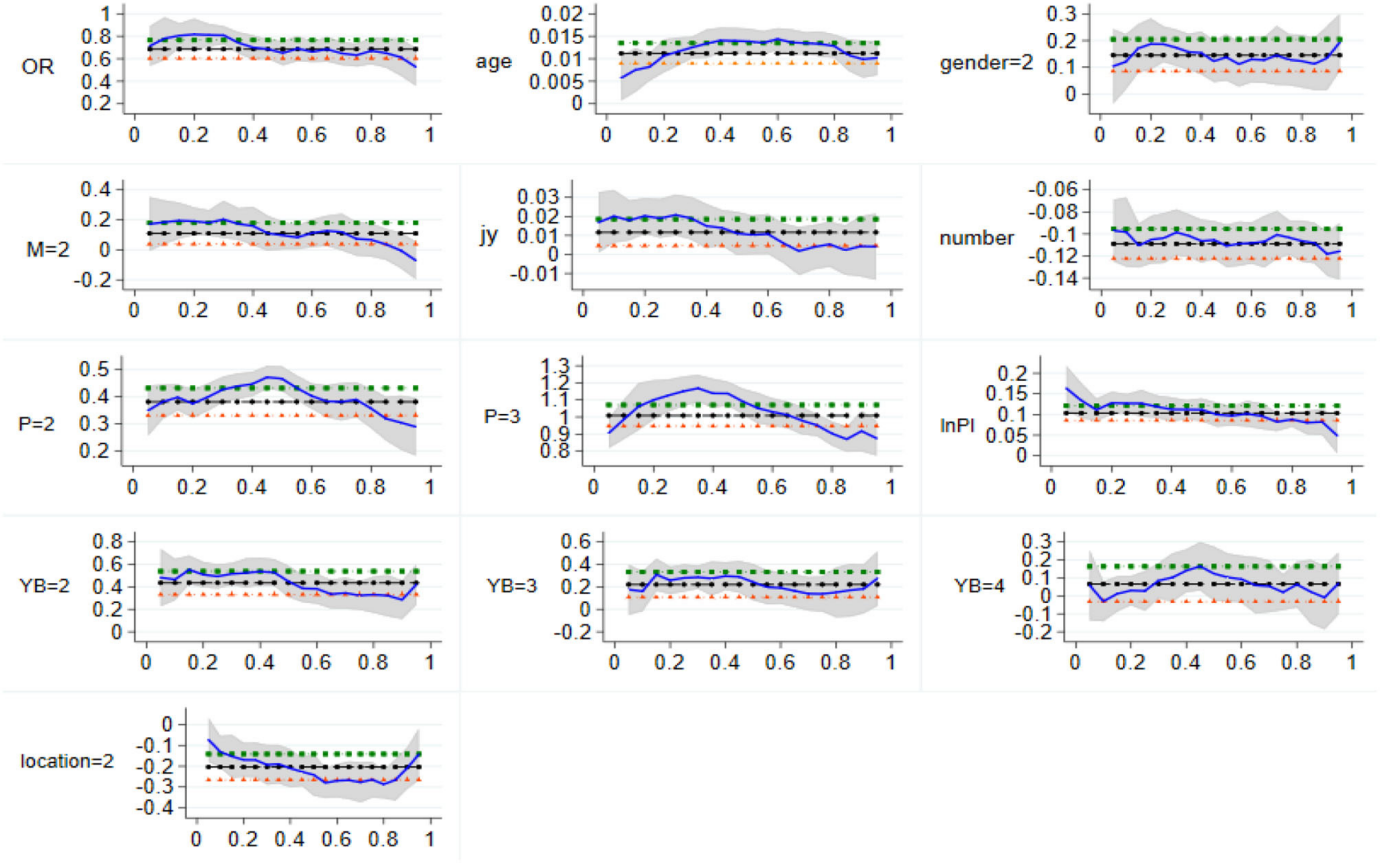
Change of quantile regression coefficient of the national sample. The vertical coordinate is the estimated value, whereas the horizontal coordinate is the different quantiles.

Based on the quantile regression results of the national sample, Figure 1 describes the changes and characteristics of the regression coefficient and confidence interval of per capita health expenditure of urban households at different quantile levels. The abscissa represents each subpoint of the per capita health expenditure of urban households, which varies isometric from 0.01 to 0.99, and the ordinate represents the regression coefficient of each explanatory variable in the regression model. It can be seen from Figure 1 that the blue curve describes the variation characteristics of the estimated coefficients of explanatory variables at different quantile levels, and the gray area represents the confidence intervals of the coefficients of each explanatory variable. In addition, for the three lines, the black dotted line in the middle represents the coefficient estimation of each explanatory variable in the OLS regression model, and the green and orange-red dotted lines on both sides represent the 95% confidence interval of each coefficient in the OLS regression model. It can be seen from the observation that, at both ends of the conditional distribution of the explained variables, the confidence interval of the estimated coefficients of each explanatory variable gradually widens, which indicates that the standard deviation of the estimated coefficient is gradually increasing, and the volatility of the estimated coefficient is constantly increasing.

#### Eastern China

The quantile regression results of the study on influencing factors of urban family health expenditure in eastern China are shown in Table 4.

**Table 4:** Regression results of household health expenditure in eastern China.

**Variables**	**QR_10**	**QR_25**	**QR_50**	**QR_75**	**QR_90**
OR	0.895^***^	0.927^***^	0.515^***^	0.639^***^	0.707^***^
	(0.118)	(0.095)	(0.090)	(0.093)	(0.123)
age	0.003	0.010^***^	0.015^***^	0.015^***^	0.008^**^
	(0.003)	(0.003)	(0.003)	(0.003)	(0.003)
gender_2	0.207^**^	0.210^***^	0.065	0.084	0.067
	(0.084)	(0.062)	(0.065)	(0.071)	(0.074)
M_2	0.262^***^	0.219^***^	0.132^*^	0.054	0.043
	(0.094)	(0.079)	(0.078)	(0.078)	(0.118)
jy	0.009	0.016^**^	0.006	0.004	0.005
	(0.010)	(0.008)	(0.008)	(0.008)	(0.010)
number	−0.116^***^	−0.092^***^	−0.126^***^	−0.104^***^	−0.117^***^
	(0.020)	(0.018)	(0.015)	(0.016)	(0.020)
P_2	0.358^***^	0.423^***^	0.428^***^	0.343^***^	0.355^***^
	(0.064)	(0.063)	(0.055)	(0.061)	(0.067)
P_3	1.057^***^	1.172^***^	1.108^***^	0.960^***^	0.880^***^
	(0.100)	(0.071)	(0.069)	(0.066)	(0.104)
lnPI	0.068^***^	0.110^***^	0.096^***^	0.066^***^	0.087^***^
	(0.022)	(0.020)	(0.023)	(0.021)	(0.029)
YB_2	0.514^***^	0.465^***^	0.366^**^	0.232^*^	0.106
	(0.120)	(0.130)	(0.151)	(0.118)	(0.172)
YB_3	0.234	0.257^**^	0.188	0.120	0.097
	(0.154)	(0.129)	(0.159)	(0.128)	(0.166)
YB_4	−0.237^**^	−0.081	−0.005	−0.105	−0.202
	(0.113)	(0.120)	(0.147)	(0.111)	(0.166)
location_2	0.065	−0.138^*^	−0.276^***^	−0.334^***^	−0.195^**^
	(0.081)	(0.077)	(0.072)	(0.074)	(0.087)
Constant	3.568^***^	3.520^***^	4.906^***^	6.448^***^	7.601^***^
	(0.298)	(0.287)	(0.312)	(0.283)	(0.403)
Observations	11,018	11,018	11,018	11,018	11,018

Robust standard errors in parentheses ^***^p < 0.01, ^**^p < 0.05, ^*^p < 0.1.

*The numbers in brackets below the regression coefficients represent robust standard error*.

As can be seen from Table 4, the main influencing factors of urban health expenditure in eastern China are the old-age dependency ratio, family size, self-rated health status, and income. The above three variables all significantly affect the health expenditure condition distribution.

First, the effect of the old-age dependency ratio on per capita health expenditure is 89.5%, larger than the national sample of 80.4%, indicating that families with low health expenditure in eastern China are more affected by population aging than the national average. The influence of control variables, such as gender, marriage, family size, and demand variables, such as self-rated health status, on dependent variables was similar to that of the national sample, and there was no abnormal situation. Meanwhile, the regression coefficient between per capita household health expenditure and per capita household income was 0.068, lower than 0.113 of the national sample. The possible reason is that the economic level of the eastern region is leading in the whole country, so the health expenditure of urban families is less affected and constrained by the income parity. Compared with the sample without any social medical insurance, the per capita health expenditure of the families of NRCMS participants is lower, indicating that these families have received the benefits of NRCMS and reduced their family health expenditure.

Second, health expenditure with middle-level health expenditure is significantly correlated with old-age dependency ratio, age, marriage, family size, self-rated health status, per capita household income, and residence. Among them, the effect of the old-age dependency ratio on per capita health expenditure reaches the minimum at this level. Every increase in the old-age dependency ratio by one unit can only increase per capita health expenditure by 51.5%, which is far less than the regression coefficients of 89.5 and 92.7% at 0.1 and 0.25 subpoints. It shows that population aging has the least influence on the average health expenditure per family in eastern China. The greater impact of residence on per capita health expenditure may be related to the gradual improvement of family health expenditure. With the increasing demand of family for medical and health services, the influence of medical resources and professional services on family health expenditure is also increasing. The promotion effect of social medical insurance on the per capita health expenditure of the family is gradually decreasing. The same reason is that the improvement in the family health expenditure makes the medical and health service become the necessary consumption expenditure of the family. Therefore, the influence of other factors becomes smaller.

**Figure 2:**
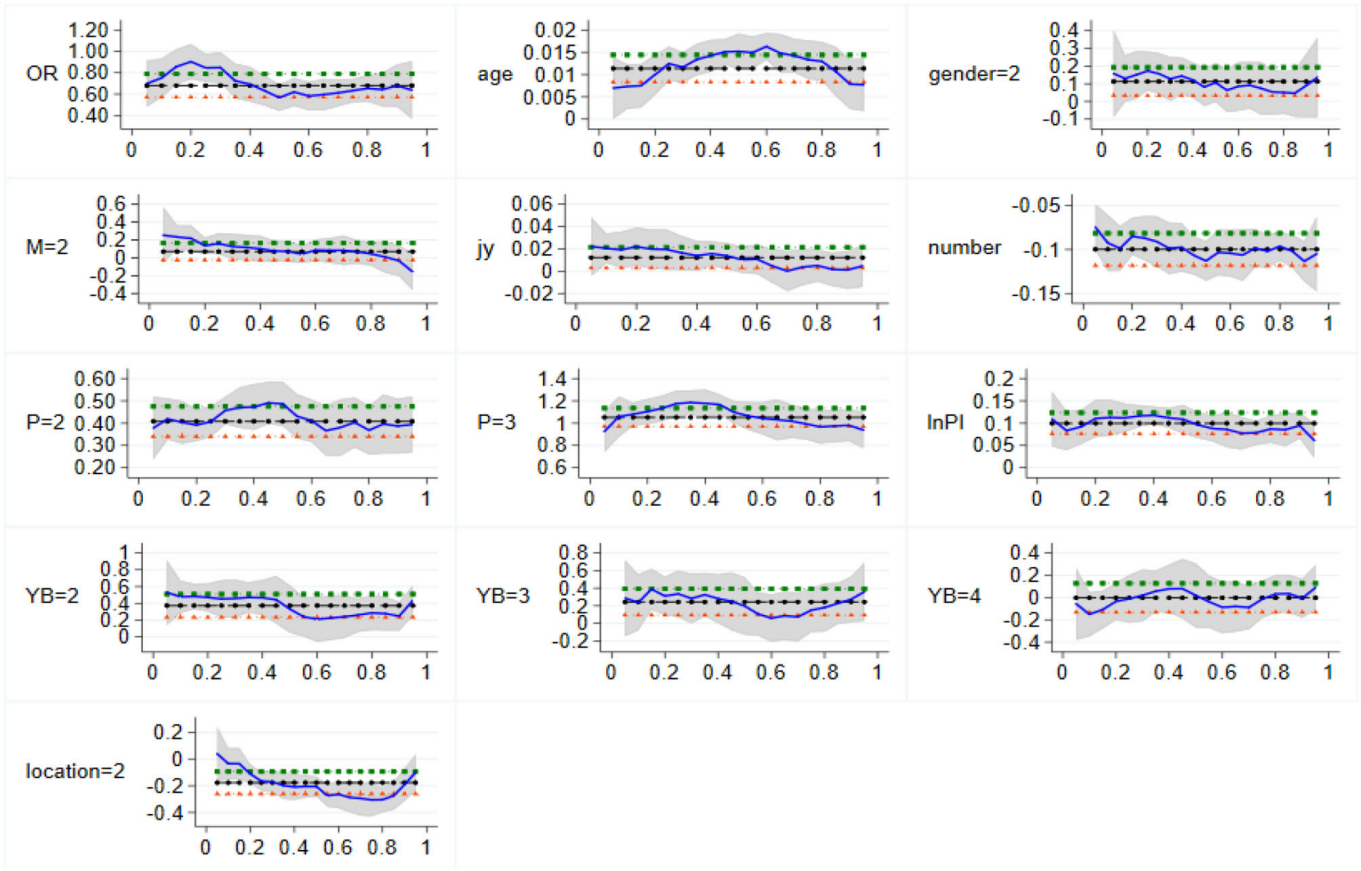
Variation of quantile regression coefficients in Eastern China. The vertical coordinate is the estimated value, whereas the horizontal coordinate is the different quantiles.

To sum up, the eastern region urban family medical expenditure on health-related factors influences the effect of the size and significance as the family per capita health spending sample conditional distribution varies. Figure 2 shows the eastern region of the changing trends and characteristics of quantile regression coefficients, which helps in a more intuitive understanding of regression results and characteristics. It shows that it has a stable influence on the sample of the condition distribution of the explained variables, can be an important factor that affects urban family medical and health care in the eastern region, and plays an important role in the development of health service and healthcare system in the eastern region.

#### Central China

The regression results of the central region are shown in Table 5.

**Table 5:** Regression results of household health expenditure in the central region.

**Variables**	**QR_10**	**QR_25**	**QR_50**	**QR_75**	**QR_90**
OR	0.714^***^	0.840^***^	0.756^***^	0.625^***^	0.619^***^
	(0.121)	(0.139)	(0.119)	(0.108)	(0.183)
age	0.000	0.011^***^	0.012^***^	0.015^***^	0.013^**^
	(0.002)	(0.003)	(0.003)	(0.003)	(0.006)
gender_2	0.180^*^	0.267^***^	0.348^***^	0.353^***^	0.262^**^
	(0.097)	(0.100)	(0.088)	(0.072)	(0.131)
M_2	0.039	0.255^**^	0.130	0.244^**^	0.185
	(0.063)	(0.100)	(0.110)	(0.104)	(0.208)
jy	0.009	0.012	0.010	−0.016	−0.004
	(0.009)	(0.012)	(0.010)	(0.010)	(0.015)
number	−0.116^***^	−0.110^***^	−0.112^***^	−0.110^***^	−0.123^***^
	(0.017)	(0.021)	(0.020)	(0.022)	(0.032)
P_2	0.366^***^	0.290^***^	0.352^***^	0.299^***^	0.077
	(0.056)	(0.088)	(0.077)	(0.074)	(0.122)
P_3	0.956^***^	1.084^***^	1.004^***^	0.806^***^	0.608^***^
	(0.111)	(0.109)	(0.088)	(0.088)	(0.145)
lnPI	0.164^***^	0.142^***^	0.131^***^	0.147^***^	0.120^***^
	(0.027)	(0.030)	(0.018)	(0.033)	(0.036)
YB_2	0.200^**^	0.369^***^	0.634^***^	0.302	−0.029
	(0.092)	(0.130)	(0.196)	(0.263)	(0.186)
YB_3	−0.154	−0.024	0.257	0.024	−0.275
	(0.127)	(0.142)	(0.202)	(0.264)	(0.221)
YB_4	−0.226^***^	−0.223^*^	0.239	0.047	−0.501^***^
	(0.066)	(0.126)	(0.191)	(0.259)	(0.154)
location_2	−0.173^**^	−0.099	−0.181^*^	−0.233^***^	−0.154
	(0.068)	(0.099)	(0.102)	(0.090)	(0.136)
Constant	3.157^***^	3.291^***^	4.275^***^	5.468^***^	7.253^***^
	(0.324)	(0.385)	(0.347)	(0.458)	(0.525)
Observations	4,439	4,439	4,439	4,439	4,439

Robust standard errors in parentheses^***^ p < 0.01, ^**^ p < 0.05, ^*^ p < 0.1.

*The numbers in brackets below the regression coefficients represent robust standard error*.

As can be seen from Table 5, factors that significantly impact each subpoint of urban family health expenditure in central China include the old-age dependency ratio, gender, family size, and per capita income. Significantly different from the regression results of the national sample and the eastern region, the effect of the old-age dependency ratio on per capita health expenditure was stable at each subsite with less change. From 0.1 subsite to 0.9 subsite, the regression coefficients of old-age dependency ratio were 0.714, 0.840, 0.756, 0.625, and 0.619, respectively. In addition, similar to the eastern region, the correlation coefficient between families with lower health expenditure and the old-age dependency ratio is the largest. Although there is no large regression coefficient of 0.927 in the eastern region, there is no significant difference in the regression coefficient of the old-age dependency ratio in the eastern region. The increase in age does not necessarily lead to an increase in health expenditure per capita in households with low levels of health expenditure, which may be due to the improvement of living standards of the population, the gradual increase in health awareness, and the improvement of diet and health habits. As a result, physical fitness has improved greatly, so increasing age does not necessarily mean increased demand for health services. Different from the regression results of the first two parts, gender has become a significant variable in the conditional distribution of explained variables in central China. The regression coefficient increases significantly, indicating that the female head of the household in central China can greatly promote family health expenditure. At the same time, the education level has become a completely insignificant variable. That is, there is no correlation between the health expenditure of urban families and the level of education of residents in central China.

**Figure 3:**
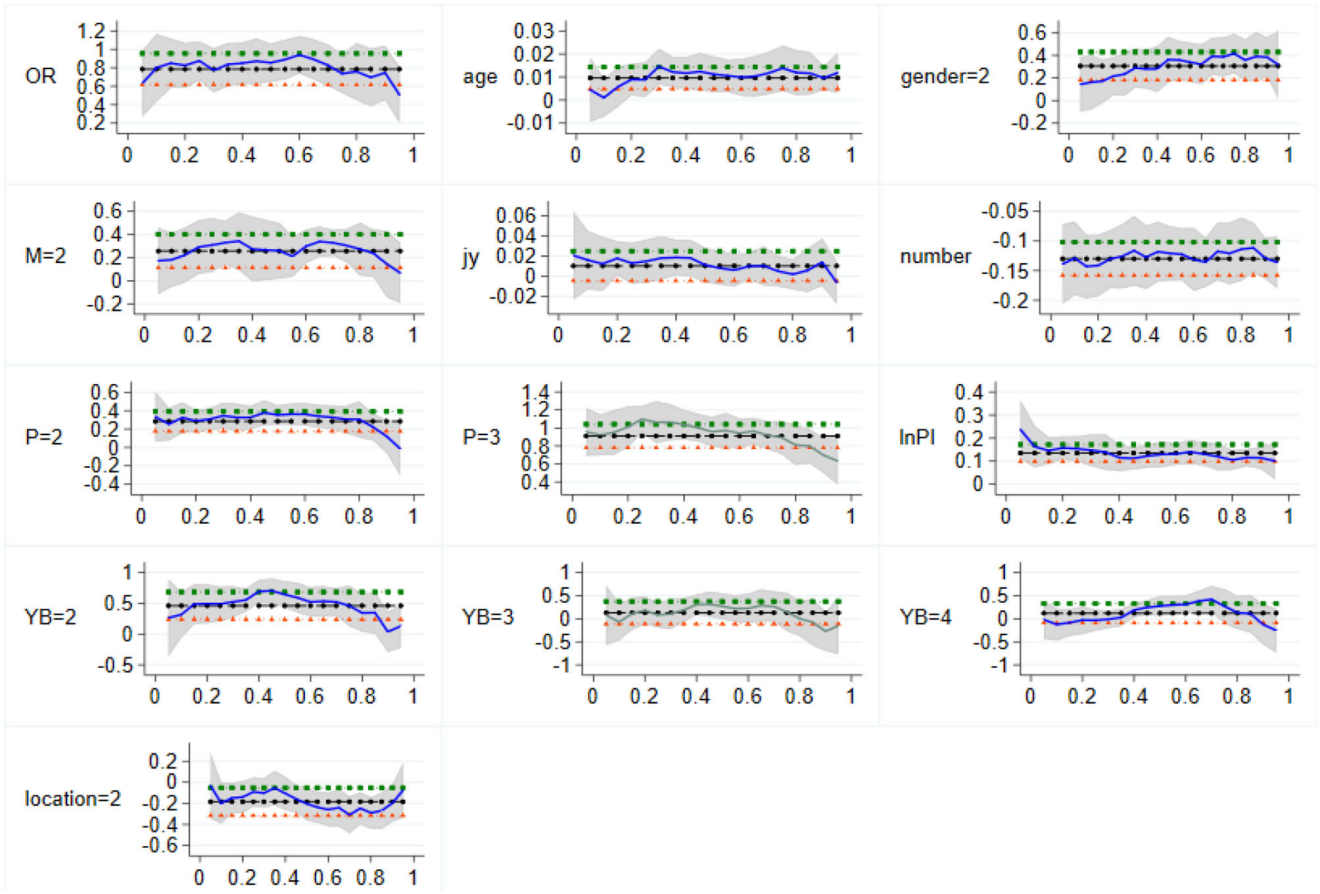
Variation of sample quantile regression coefficients in Central China. The vertical coordinate is the estimated value, whereas the horizontal coordinate is the different quantiles.

At the same time, similar to the influence of residence in the eastern region, the residence in the central region has the greatest influence on the per capita health expenditure of families with high health expenditure, with a regression coefficient of 0.233, lower than 0.334 in the eastern region. This may be because the medical and health resources and professionals in the eastern region are richer than those in the central region, so the medical cost of urban families in the eastern region is higher, and the use of medical and health services is more convenient and comprehensive. Thus, the health expenditure of urban families in the eastern region is higher. Refer to Figure 3 for the specific variation characteristics of the regression coefficients of each variable.

#### Western China

The regression results of the western region are shown in Table 6.

**Table 6:** Regression results of household health expenditure in western China.

**Variables**	**QR_10**	**QR_25**	**QR_50**	**QR_75**	**QR_90**
OR	0.542^***^	0.743^***^	0.513^***^	0.444^**^	0.483^***^
	(0.198)	(0.147)	(0.140)	(0.176)	(0.118)
age	0.014^***^	0.015^***^	0.015^***^	0.015^***^	0.015^***^
	(0.005)	(0.004)	(0.004)	(0.005)	(0.003)
gender_2	0.048	0.307^***^	0.062	0.103	0.033
	(0.128)	(0.100)	(0.107)	(0.113)	(0.067)
M_2	0.146	0.376^***^	0.185	−0.013	−0.064
	(0.131)	(0.117)	(0.129)	(0.150)	(0.104)
jy	0.031^**^	0.045^***^	0.025^**^	0.007	0.014
	(0.016)	(0.013)	(0.012)	(0.015)	(0.011)
number	−0.146^***^	−0.139^***^	−0.111^***^	−0.136^***^	−0.128^***^
	(0.034)	(0.028)	(0.020)	(0.028)	(0.017)
P_2	0.380^***^	0.507^***^	0.547^***^	0.328^***^	0.188^**^
	(0.120)	(0.095)	(0.091)	(0.107)	(0.077)
P_3	0.804^***^	1.130^***^	1.208^***^	0.750^***^	0.793^***^
	(0.147)	(0.121)	(0.103)	(0.136)	(0.133)
lnPI	0.106^***^	0.105^***^	0.080^**^	0.083^**^	0.013
	(0.038)	(0.037)	(0.033)	(0.039)	(0.023)
YB_2	0.925^***^	0.648^***^	0.659^***^	0.805^***^	0.598^***^
	(0.162)	(0.125)	(0.207)	(0.265)	(0.212)
YB_3	0.308	0.207	0.480^**^	0.690^**^	0.528^***^
	(0.188)	(0.209)	(0.223)	(0.278)	(0.203)
YB_4	0.657^***^	0.279^*^	0.429^**^	0.543^**^	0.506^**^
	(0.145)	(0.143)	(0.204)	(0.258)	(0.206)
location_2	−0.236^*^	−0.277^**^	−0.449^***^	−0.348^**^	−0.546^***^
	(0.140)	(0.118)	(0.112)	(0.136)	(0.107)
Constant	2.176^***^	2.796^***^	4.395^***^	5.928^***^	7.726^***^
	(0.485)	(0.467)	(0.449)	(0.555)	(0.334)
Observations	4,330	4,330	4,330	4,330	4,330

Robust standard errors in parentheses^***^ p < 0.01, ^**^ p < 0.05, ^*^ p < 0.1.

*The numbers in brackets below the regression coefficients represent robust standard error*.

According to the quantile regression results shown in Table 6, it can be seen that:

First of all, the influence of the old-age dependency ratio on the health expenditure of urban families in western China is generally weaker than that in eastern and central China. Similarly, the influence of families with a lower old-age dependency ratio on health expenditure is the largest at each subsite. In addition, the regression coefficients of the old-age dependency ratio at 0.25 subpoint in eastern, central, and western regions were 0.927, 0.840, and 0.743, respectively, which may be related to the different degrees of aging in different regions. At the same time, the robust standard error of the estimation coefficient of the old-age dependency ratio was larger than that of the eastern and central regions, and the size of the standard error was 0.198, 0.147, 0.140, 0.176, and 0.118 from the low loci to the high loci, indicating that the estimation of quantile regression coefficient at the right end of the conditional distribution was more accurate. The quantile regression coefficient of the left end of the conditional distribution is different. That is, the increase in per capita health expenditure of urban families in western China is <19.8% and close to 11.8% when the old-age dependency ratio increases by one unit.

Second, gender and marital status only have a significant positive impact on the per capita health expenditure of families with low health expenditure, while there is a positive correlation between the level of health expenditure of families with medium or lower level and residents' education level. In addition, the effects and characteristics of family size and self-rated health status on per capita health expenditure are similar to those in eastern and central China, which are both important influencing factors. At the same time, the central region of the family healthcare spending has no obvious relation with residents affected by education level. It shows that the education level of urban areas in western China is relatively backward as a whole. There is still a large space for development.

**Figure 4:**
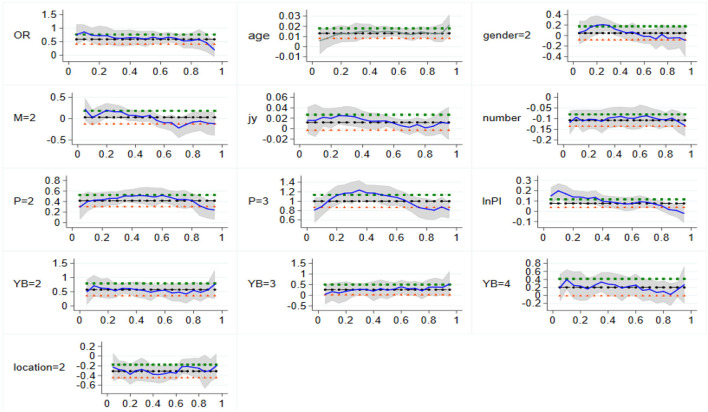
Change of quantile regression coefficient in Western China. The vertical coordinate is the estimated value, whereas the horizontal coordinate is the different quantiles.

Third, the impact of per capita household income on per capita household health expenditure shows a U-shaped trend of decline first and then rise, and families with high levels of health expenditure are not affected by per capita household income, indicating that there is a large gap in economic level between urban families in western China. Based on the analysis of the regression results and coefficients of the above influencing factors, Figure 4 shows the detailed variation trend of the quantile regression coefficients of different influencing factors of urban household health expenditure in western China. Compared with residents without any social medical insurance, urban employment insurance can significantly increase the per capita health expenditure of families at each subpoint, and with the increase of subpoints, the regression coefficients are 0.925, 0.648, 0.659, 0.805, and 0.598, respectively.

### Robustness Test

To test the robustness of the quantile regression model, this paper used elderly population coefficient (EPC, i.e., each household age 65 and older population ratio of the total population) instead of the old-age dependency ratio. The regression results of the nationwide sample are shown in Table 7.

**Table 7:** Robustness test results.

**Variables**	**QR_10**	**QR_25**	**QR_50**	**QR_75**	**QR_90**
EPC	0.925^***^	0.908^***^	0.663^***^	0.615^***^	0.656^***^
	(0.103)	(0.080)	(0.069)	(0.078)	(0.092)
age	0.005^**^	0.011^***^	0.014^***^	0.014^***^	0.011^***^
	(0.002)	(0.002)	(0.002)	(0.002)	(0.003)
gender_2	0.190^***^	0.252^***^	0.140^***^	0.148^***^	0.096
	(0.059)	(0.058)	(0.050)	(0.054)	(0.064)
M_2	0.160^***^	0.259^***^	0.114^*^	0.098	0.039
	(0.055)	(0.066)	(0.060)	(0.075)	(0.092)
jy	0.025^***^	0.022^***^	0.008	0.000	0.006
	(0.007)	(0.007)	(0.006)	(0.007)	(0.008)
number	−0.108^***^	−0.106^***^	−0.116^***^	−0.111^***^	−0.112^***^
	(0.011)	(0.014)	(0.011)	(0.013)	(0.016)
P_2	0.363^***^	0.415^***^	0.429^***^	0.330^***^	0.223^***^
	(0.046)	(0.050)	(0.042)	(0.049)	(0.053)
P_3	0.947^***^	1.138^***^	1.090^***^	0.899^***^	0.800^***^
	(0.071)	(0.059)	(0.051)	(0.056)	(0.086)
lnPI	0.106^***^	0.117^***^	0.111^***^	0.090^***^	0.075^***^
	(0.013)	(0.016)	(0.016)	(0.019)	(0.024)
YB_2	0.435^***^	0.496^***^	0.512^***^	0.348^***^	0.229^*^
	(0.113)	(0.089)	(0.113)	(0.111)	(0.121)
YB_3	0.095	0.203^*^	0.290^**^	0.149	0.156
	(0.141)	(0.105)	(0.118)	(0.119)	(0.129)
YB_4	−0.107	−0.035	0.150	0.034	−0.126
	(0.105)	(0.085)	(0.111)	(0.107)	(0.119)
location_2	−0.036	−0.144^**^	−0.273^***^	−0.298^***^	−0.242^***^
	(0.059)	(0.059)	(0.054)	(0.060)	(0.073)
Constant	3.003^***^	3.300^***^	4.596^***^	6.126^***^	7.499^***^
	(0.198)	(0.220)	(0.226)	(0.260)	(0.315)
Observations	19,787	19,787	19,787	19,787	19,787

Robust standard errors in parentheses^***^ p < 0.01, ^**^ p < 0.05, ^*^ p < 0.1.

*The numbers in brackets below the regression coefficients represent robust standard error*.

Compared with Table 3, the significance of each variable in different quantiles does not obviously change after the old-age dependency ratio is replaced by EPC in Table 7. For example, in Table 3, the old-age dependency ratio regression coefficients from low-score loci to high-score loci were 0.804, 0.856, 0.624, 0.588, and 0.626, respectively. In Table 7, the regression coefficients of EPC from 0.1 to 0.9 were 0.925, 0.908, 0.663, 0.615, and 0.656, respectively. There was a significant positive correlation between them and health expenditure. Therefore, it can be proved that the regression results of the empirical study in this paper have strong robustness. However, it is worth noting that the robust standard error of the regression estimation coefficient of the old-age dependency ratio is 0.096, 0.078, 0.067, 0.076, and 0.097, respectively, whereas the robust standard error of EPC is 0.103, 0.080, 0.069, 0.078, and 0.092, respectively. Therefore, the old-age dependency ratio is slightly more accurate in estimating the conditional distribution of the national sample. Therefore, this study chooses the old-age dependency ratio as the measurement index of population aging, rather than EPC.

## Discussion

### Main Conclusion

This paper explores the impact of the dependency burden on urban household health expenditure and its regional heterogeneity in China based on the micro-data of CHFS. A total of 19,787 valid samples were obtained and the quantile regression method was used due to the skewness of the distribution of explained variables and the possible extreme values. Some useful results were found.

First, the old-age dependency ratio, age, family size, self-rated health status, and income significantly impact the health expenditure of urban families at the national sample quantile regression [similar to Carreras et al. (27), Ross et al. (2), Hou and Ren (5), and Xie et al. (9)]. The impact of gender on health expenditure of low health expenditure families is significant. Marriage has a significant impact on the health expenditure of families with moderate and lower levels of health expenditure. This shows that individuals in marriage pay more attention to their own health problems and their impact on the family. Education level only has a positive effect on families with low health expenditure. This conclusion is further confirmed in Hjortsberg (11) and Tian et al. (14). Only the basic medical insurance for urban workers has significantly impacted health expenditure. This is basically the same as Sepehri and Vu (22), but opposite to Xu and Chen (6), which is an upward trend, the particularity of national conditions in China led to the cognitive difference in this problem.

Second, dependency burden and other variables on urban household health expenditure have great regional heterogeneity. For instance, the old-age dependency ratio has a significant effect on the per capita health expenditure; however, it is most obvious in eastern China [similar to Li et al. (28)]. This may have much to do with the cost of providing for the aged in eastern China. Due to the higher overall economic level, medical expenses and other pension costs in eastern China are much higher than those in western and central China. In addition, gender has the most significant effect on health expenditure in central China. In contrast, marriage and education level have a different effect on health expenditure in all the three regions [consistent with Mackenbach et al. (13)]. Family size, self-rated health status, and income have significant and stable effects on each region. Social medical insurance for the influence of various regions is more complicated. In addition, the relationship between social medical insurance and health expenditure shows more closely in western China (23, 25) reached the same conclusion. This may be related to the low economic level of western China, the lower the income, the stronger the dependence on medical insurance.

### Policy Implications

The above conclusions can help us put forward some policy recommendations.

First, government agencies should unite with all social strata to establish a multi-level social security system for aged care and formulate relevant policies to establish a medical care system for the older adult population suitable for economic development as soon as possible.

Second, the aging population greatly affects health expenditure in eastern China. This reflects the higher level of economic development in the eastern region, which leads to the early emergence of a low birth rate, low natural growth rate, and low death rate in the population structure of the eastern region, and the aging degree is deep. The aging problem in the eastern region cannot be improved significantly in the short term, so the medical institution service system related to the older adult population in the eastern region should be improved as soon as possible to ensure that the medical needs and old-age services of the elderly can be satisfied.

Third, the correlation between the urban health expenditure and their residence is the most stable and strong in western China. The differences in residential areas represent the lack of medical and health resources such as medical institutions and medical professionals between urban and rural families. Therefore, we should pay attention to the introduction of professional medical personnel and the configuration of precision medical equipment in the western region to improve the medical and health system in the western region.

## Conclusion

There are some improvements in this study. For example, this paper selects the old-age dependency ratio as the core explanatory variable such as the fiscal policy factor (32, 33), which may omit other important variables that have not been paid attention to. Thus, the selection of research indicators is bound to have greater progress and improvement space in the future. From the sample data, this paper only uses the cross-sectional data of CHFS in 2017, while explanatory variables such as family health expenditure and population aging are dynamic processes and may show different characteristics in different time periods. Therefore, to a certain extent, the research in this paper cannot reflect the characteristics and trends of family medical expenses in different years, and the conclusions may have some limitations. All these need to be further improved in the future.

## Data Availability Statement

The original contributions presented in the study are included in the article/supplementary material, further inquiries can be directed to the corresponding author/s.

## Ethics Statement

The data of this study came from China Household Finance Survey (CHFS) datasets (publicly available datasets). All methods were carried out in accordance with relevant guidelines and regulations (Declaration of Helsinki).

## Author Contributions

XX: conceptualization, writing, and editing. QW: methodology and software. CL: data curation and project administration. All authors contributed to the article and approved the submitted version.

## Funding

This paper is a phased achievement of The National Social Science Fund of China: Research on the blocking mechanism of the critical poor households returning to poverty due to illness, no: 20BJY057.

## Conflict of Interest

The authors declare that the research was conducted in the absence of any commercial or financial relationships that could be construed as a potential conflict of interest.

## Publisher's Note

All claims expressed in this article are solely those of the authors and do not necessarily represent those of their affiliated organizations, or those of the publisher, the editors and the reviewers. Any product that may be evaluated in this article, or claim that may be made by its manufacturer, is not guaranteed or endorsed by the publisher.
